# Poor sleep quality among patients with Parkinson’s disease: a meta-analysis and systematic review

**DOI:** 10.3389/fpsyt.2025.1606743

**Published:** 2025-07-16

**Authors:** Tong Leong Si, Yue-Ying Wang, Jia-Xin Li, Wei Bai, He-Li Sun, Shu-Ying Rao, Han-Yu Zhu, Gabor S. Ungvari, Zhaohui Su, Teris Cheung, Chee H. Ng, Yu-Tao Xiang, Gang Wang

**Affiliations:** ^1^ Macao Observatory for Social Development, University of Saint Joseph, Macao, Macao SAR, China; ^2^ Unit of Psychiatry, Department of Public Health and Medicinal Administration, Institute of Translational Medicine, Faculty of Health Sciences, University of Macau, Macao, Macao SAR, China; ^3^ Centre for Cognitive and Brain Sciences, University of Macau, Macao, Macao SAR, China; ^4^ Department of Epidemiology and Biostatistics, School of Public Health, Jilin University, Changchun, China; ^5^ Section of Psychiatry, University of Notre Dame Australia, Fremantle, WA, Australia; ^6^ Division of Psychiatry, School of Medicine, University of Western Australia, Perth, WA, Australia; ^7^ School of Public Health, Southeast University, Nanjing, China; ^8^ School of Nursing, Hong Kong Polytechnic University, Hong Kong, Hong Kong SAR, China; ^9^ Department of Psychiatry, The Melbourne Clinic and St Vincent’s Hospital, University of Melbourne, Richmond, VIC, Australia; ^10^ Beijing Key Laboratory of Mental Disorders, National Clinical Research Center for Mental Disorders & National Center for Mental Disorders, Beijing Anding Hospital, Capital Medical University, Beijing, China

**Keywords:** Parkinson’s disease, sleep quality, meta-analysis, prevalence, epidemiology

## Abstract

**Objective:**

Poor sleep quality is common among patients with Parkinson’s disease (PD), although the reported prevalence rates vary between studies. This meta-analysis examined the overall prevalence of poor sleep quality in patients with PD and identified potential factors contributing to the differences in prevalence across studies.

**Methods:**

Both PRISMA and MOOSE guidelines were applied in this meta-analysis. A systematic search was conducted in PubMed, EMBASE, PsycINFO, Web of Science, CNKI and Wangfang from their inception to November 4, 2023. Studies were selected based on predefined PICOS criteria (i.e., PD patients, prevalence of poor sleep quality, cross-sectional/cohort designs). Study quality/risk of bias was assessed using a standardized 8-item tool. Pooled prevalence was calculated sources of heterogeneity (e.g., age, sex, depression, anxiety, cognition scores, disease severity, and medication dose) were explored via subgroup and meta-regression analyses. A random-effects model was utilized to calculate the overall prevalence and corresponding 95% confidence intervals (CIs).

**Results:**

In total, 63 studies involving 9,382 PD patients were included. The overall prevalence of poor sleep quality was 58.07% (95% CI: 54.26–61.88%). Higher rates were related to various factors including studies from Europe & Central Asia, Upper middle income countries, mixed patient sources, lower diagnostic cutoffs, and use of Movement Disorder Society PD criteria. Meta-regression analysis showed that late onset PD was associated with poorer sleep quality in patients with PD.

**Conclusion:**

Poor sleep quality is common in PD patients. Regular monitoring of sleep quality and promotion of sleep hygiene should be prioritized in the management of patients with PD. Additionally, further research on sleep and PD is warranted in low- and middle-income countries to ensure the applicability of findings across diverse populations.

**Systematic Review Registration:**

https://inplasy.com/inplasy-2023-10-0022/, identifier INPLASY2023100022.

## 1 Introduction

Parkinson’s disease (PD) is a progressive neurological disorder that interferes with the initiation and execution of voluntary movement, resulting in difficulty performing basic activities of daily living (1). PD is an age-related condition as its prevalence steadily increases with age (2). Although the cause is still unknown, many researchers believe that the disease is determined by the interaction between genetic and environmental factors, leading to a gradual degeneration of neurons in susceptible areas of the brain (2). Apart from the typical motor symptoms, non-motor symptoms in patients with PD, including sleep problems, autonomic dysfunction and sensory abnormalities (3), also have a significant impact on quality of life and overall health (4, 5).

Sleep problems in PD patients, such as poor sleep quality, are among the most prominent non-motor symptoms that considerably impact on the quality of life in both patients and caregivers (6, 7). The causes of sleep problems in PD patients involve disease-related factors such as dopaminergic medications, co-morbid mood disorders, and other related factors (8), as well as the associated symptoms and treatments of PD (9).

Poor sleep quality is one of the major sleep problems experienced by PD patients (10). Sleep quality is defined as one’s satisfaction with sleep experience, encompassing aspects such as sleep onset, sleep maintenance, subjective sleep quality, and mental state upon awakening (11). Both subjective and objective methods can be employed to assess sleep quality. Objective techniques usually have a high reliability in collecting sleep parameter data, such as using actigraphy and polysomnography (12). However, these objective sleep quality tests are costly, time-consuming, not easily accessible by most practitioners, and unsuitable for use in epidemiological studies and clinical practice (13). As such, subjective measures on sleep quality have been developed for such purposes, for instance, the Pittsburgh Sleep Quality Index (PSQI). The PSQI is the most utilized subjective measurement of sleep quality across different populations, providing scores that allow for categorization as “good” or “poor” (14, 15). In addition, the Parkinson’s Disease Sleep Scale (PDSS) is another widely used visual analog scale that addresses several symptoms associated with sleep disorders in PD, including sleep quality (16).

Previous studies on the prevalence of poor sleep quality in patients with PD have reported rates ranging from 20% to 88% (17–19). In addition, poor sleep quality in PD patients has been found to be associated with more severe comorbid depressive and anxiety symptoms, reduced cognitive performance, and more severe PD symptoms (20). Therefore, to address the negative impact of poor sleep quality on PD patients, it is important to understand the epidemiological patterns of poor sleep quality and its associated factors in this population.

To date, no systematic review and meta-analysis have been published on the prevalence estimates of poor sleep quality in PD patients. To address this gap, this meta-analysis aimed to assess the global prevalence of poor sleep quality in PD patients and identify potential factors contributing to the differences in prevalence in this population.

## 2 Method

### 2.1 Search strategy

The Preferred Reporting Items for Systematic Reviews and Meta-Analyses (PRISMA) guidelines and Meta-analyses Of Observational Studies in Epidemiology (MOOSE) checklist were followed in conducting this meta-analysis (21). Three investigators (TLS, YYW and JXL) systematically and independently searched literature in the PubMed, EMBASE, PsycINFO, Web of Science, China National Knowledge Infrastructure (CNKI) and Wangfang databases from their inception date until November 4, 2023, using the following search items: “Parkinson disease “ AND (“Sleep Quality” OR “Qualities, Sleep” OR “Quality, Sleep” OR “Sleep Qualities” OR “quality of sleeping” OR “sleeping quality” OR “Pittsburgh sleep quality index” OR “PSQI”) AND (“prevalence” OR “epidemiology” OR “rate”). The study protocol was registered with the International Platform of Registered Systematic Review and Meta-analysis Protocols (INPLASY; registration number: INPLASY2023100022).

### 2.2 Inclusion and exclusion criteria

The same three investigators (TLS, YYW and JXL) independently reviewed the titles and abstracts of relevant publications and then read the full texts of to assess eligibility. The inclusion criteria were developed using the PICOS acronym: Participants (P): patients with PD according to study-defined diagnostic criteria, such as the UK PD Society Brain Bank criteria (22) and the Movement Disorder Society (MDS) clinical diagnostic criteria for PD (23); Intervention (I): not applicable; Comparison (C): NR; Outcome (O): prevalence of poor sleep quality or having information that could generate an estimation of that prevalence, using standard tools, such as the PSQI, to measure the quality of the sleep; Study design (S): cross-sectional and cohort studies (only baseline data were analyzed in cohort studies) with accessible data published in English or Chinese journals. Exclusion criteria included reviews, systematic reviews, meta-analyses, case studies, and commentaries. Further, studies conducted specifically on PD samples with a primary sleep disorder (e.g., PD patients recruited from sleep clinics who suffered from insomnia disorder or obstructive sleep apnea (OSA)) were excluded to avoid the risk of overrepresenting sleep disturbances and selection bias in PD samples (24, 25). Only the study with the most detailed information was included in the meta-analysis if a dataset was utilized in more than one study (26).

### 2.3 Data extraction and quality assessment

Participant and study data, such as the first author, publication year, sampling method, sleep quality measures, number of PD patients, cut-off value, illness duration, number of participants with poor sleep quality, and sleep quality scores, mean age and male proportion of study sample, diagnostic criteria, were extracted. Additionally, to characterize the multidimensional aspects of sleep, we extracted specific components from standardized sleep tools (e.g., the PSQI domains: subjective sleep quality, sleep latency, sleep duration, habitual sleep efficiency, sleep disturbance, use of sleep medications, and daytime dysfunction).

Such components including sleep medication use could enable the evaluation of naturally occurring sleep within broadly representative PD cohorts, rather than selective studies that focused on sleep comorbidities and effects of treatment.

Study quality was assessed using a standardized instrument for epidemiological studies (27, 28) with the following eight items: (1) target population was defined clearly; (2) probability sampling or entire population surveyed; (3) response rate was ≥80%; (4) non-responders were clearly described; (5) sample was representative of the target population; (6) data collection methods were standardized; (7) validated criteria were used to diagnose PD; and (8) prevalence estimates were given with confidence intervals (CIs) and detailed by subgroups. The total score ranged from 0 to 8. Studies with a total score of “7–8” were considered as “high quality,” “4–6” as “moderate quality,” and “0–3” as “low quality” based on previous studies (25, 29, 30).

### 2.4 Statistical analyses

All the statistical analyses were conducted using R program (31). A random-effects model was used to synthesis the pooled prevalence of poor sleep quality and its 95% confidence intervals (95% CI) (32). *I^2^* statistics were used to determine the degree of study heterogeneity, with 25%, 50%, and 75% tentatively indicating low, moderate, and high heterogeneity, respectively (33). Subgroup analyses for categorical variables (e.g., study regions, countries by economic status according to the World Bank’s criteria (34), study design, cut-off value signifying poor sleep quality, and patients resources), and meta-regression analysis for continuous variables (mean age, proportions of male, depression, anxiety and cognitive dysfunction respectively, mean scores of MoCA, mean scores of MMSE and mean scores of Hoehn and Yahr Staging, mean levodopa equivalent daily dose and quality assessment score) were conducted to explore the sources of potential heterogeneity. The Egger’s test was used to evaluate publication bias, and a visual funnel plot for asymmetry was also provided. If there was publication bias (*P*<0.05), a trim-and-fill analysis was used. Sensitivity analyses were carried out to assess the stability of results by individually eliminating each study. The significance level was set at 0.05 (two-tailed).

## 3 Results

### 3.1 Search results and study characteristics

**Figure 1:**
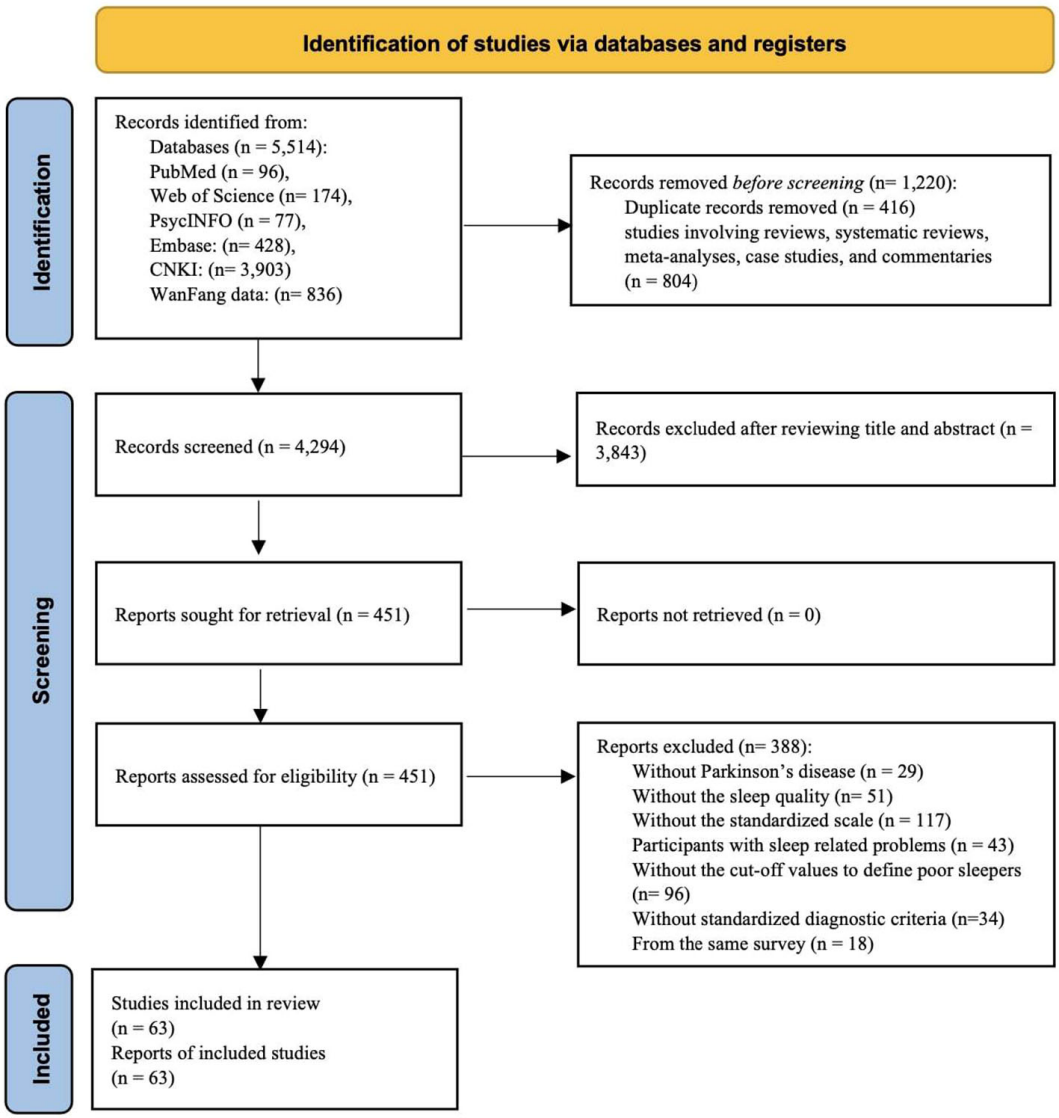
PRISMA flow diagram of study selection.

Out of a total of 5,514 publications initially retrieved, 1,220 were excluded. The remaining 4,294 studies were screened for eligibility. By screening the titles and abstracts, we identified 451 potentially eligible studies. After screening the full text of these studies, we ultimately included 63 studies in this meta-analysis (see **Figure 1**).

As shown in **Table 1**, 63 studies covering a total of 9,382 participants were included, with the sample sizes ranging from 40 to 938. The mean age of participants ranged from 51.42 to 74.97 years, the mean disease duration ranged from 0.56 to 9 years, and the mean age at onset ranged from 48.49 to 64.57 years. Regarding the use of scales for sleep quality in the PD patients, 58 studies used the PSQI, 2 studies used the PDSS, 2 studies used the PDSS-1 (the first entry in the Parkinson’s Disease Sleep Scale), and 2 studies used the PDSS-2 (Parkinson’s Disease Sleep Scale-2). We applied quality assessment on all 63 studies, where one was rated “low quality” (1.89%), while 62 studies were rated as “moderate quality” (98.41%), with study quality assessment scores ranging from 3 to 6 (See **Supplementary Table S1**).

**Table 1:** Characteristics of studies included in the meta-analysis.

Author and Reference Number	Study site	Study design	Sample size	Scale	Cut-off	Mean age ± SD (Year)	Mean disease duration ± SD (Year)	Mean onset age ± SD (Year)	Diagnostic criteria	Mean MoCA total scores ± SD	Mean MMSE total scores ± SD	Mean UPDRS-3 scores ± SD	Mean LEDD ± SD (mg/d)	Mean H&Y stage ± SD	Study quality score
Ma and Ren (76)	China	cross-sectional	110	PSQI	>4	63.2	4.1	NR	UKPDS	21.22 ± 4.22	NR	NR	NR	NR	5
Chen, Zhang, Zhou, Yang and Feng (77)	China	cross-sectional	60	PSQI	>4	63.08 ± 11.93	6.33 ± 3.73	NR	UKPDS	NR	NR	NR	NR	NR	4
Chen, Hu, Han, Zhang and Zhang (78)	China	cross-sectional	48	PSQI	>5	NR	NR	NR	PD criteria by NEDC	26.54 ± 3.85	NR	33.93 ± 19.82	NR	4.17	4
Chen, Hong and Chen (79)	China	cross-sectional	265	PSQI	>7	63.1 ± 8.8	4.35 ± 2.12	NR	UKPDS	24.53 ± 3.64	NR	20.51 ± 10.01	NR	NR	4
Guo, Yu, Zuo, Liu, Li, Pu, Hu, Lian, Wang, Yu, Jin, Zhu and Zhang (80)	China	cross-sectional	94	PSQI	>4	65.08 ± 2.23	5.15 ± 1.3	59.93 ± 2.01	UKPDS	21.58 ± 1.18	26.55 ± 0.94	19.09 ± 2.33	NR	NR	4
Guo (81)	China	cross-sectional	100	PSQI	>7	74.97 ± 6.84	8.87 ± 4.3	NR	UKPDS	NR	NR	NR	NR	NR	4
Yuan, Hu, Zhao, Wang, Zhang and Li (82)	China	cross-sectional	83	PSQI	>7	69.04 ± 9.69	5.05 ± 3.4	63.99 ± 9.69	Diagnostic criteria for PD in China	NR	NR	27.94 ± 18.17	NR	NR	4
Xue (83)	China	cross-sectional	50	PSQI	>6	72.36 ± 5.69	NR	NR	Diagnostic criteria for PD in China	NR	NR	NR	NR	NR	4
Hu and Zhang (84)	China	cross-sectional	170	PSQI	>6	65.78 ± 9.1	4.08 ± 3.34	NR	UKPDS	NR	28.47 ± 1.64	16.3 ± 8.48	400 ± 210	2.12 ± 1.25	4
Cheng, Yu and Wu (85)	China	cross-sectional	95	PSQI	>7	52.53 ± 14.71	NR	NR	Diagnostic criteria for PD in China	NR	NR	NR	NR	NR	4
Wang, Feng, Gu, Liu and Chen (86)	China	cross-sectional	486	PSQI	>6	62.52 ± 10.03	4.42 ± 3.69	58.18 ± 10.41	UKPDS	NR	27.33 ± 2.89	NR	NR	NR	5
Wang, Feng, Gu, Liu, Zhang and Chen (87)	China	cross-sectional	535	PSQI	>6	64.24 ± 8.05	4.39 ± 3.86	59.91 ± 8.76	UKPDS	NR	27.17 ± 2.89	21.01 ± 12.27	NR	2.04 ± 1.84	5
Wang and Wang (88)	China	cross-sectional	45	PSQI	>4	64.07 ± 10.1	2.66 ± 2.22	61.41 ± 10.18	UKPDS	22.66 ± 5.53	NR	NR	NR	NR	5
Wang, Sun, Ma, Shi, Li, Huang, Hu and Zheng (89)	China	cross-sectional	359	PSQI	>6	62.84 ± 7.81	6.25 ± 4.56	NR	Diagnostic criteria for PD in China	NR	NR	38.19 ± 28.9	NR	NR	4
Mao and Zhang (90)	China	cross-sectional	112	PSQI	>7	65.06 ± 6.49	6.39 ± 3.09	NR	Diagnostic criteria for PD in China	21.83 ± 4.86	NR	40.05 ± 14.21	NR	1.67	4
Mao, Dai and Liu (91)	China	cross-sectional	53	PSQI	>5	62.17 ± 9.26	4.91 ± 3.78	57.7 ± 8.43	UKPDS	NR	NR	NR	NR	1.8 ± 0.4	4
Mao and Saimaiti (92)	China	cross-sectional	46	PSQI	>5	NR	NR	NR	PD criteria by NEDC	28.11 ± 2.91	NR	32.91 ± 16.13	NR	NR	3
Liang, Cui, Wu, Yu and Chen (93)	China	cross-sectional	120	PSQI	>6	62.73 ± 8.85	5.87 ± 2.87	NR	UKPDS	NR	NR	NR	NR	NR	4
Zhu, Zheng and Yang (94)	China	cross-sectional	58	PSQI	>5	65.07 ± 8.1	5.26 ± 2.5	NR	PD criteria by NEDC	NR	NR	NR	NR	1.6 ± 1.02	5
Cao, Liu, Ma, Chen, Ren, Zhang and Xu (95)	China	cross-sectional	75	PSQI	>6	63.2 ± 10.26	3.11 ± 2.92	58.24 ± 12.58	MDS-PD	19.53 ± 5.69	24.41 ± 4.80	31.93 ± 14.05	NR	1.85 ± 1.20	4
Cao, Yu, Su and Guo (96)	China	cross-sectional	93	PSQI	>5	67.58 ± 8.14	3.01 ± 2.74	64.57 ± 8.67	UKPDS	NR	26.44 ± 1.03	NR	NR	2.47 ± 0.98	4
Zhang, Gao and Wei (97)	China	cross-sectional	68	PSQI	>6	61.23 ± 7.1	3.78 ± 3.12	NR	UKPDS	NR	NR	NR	NR	NR	4
Zhang, Zhang, Zhu, Jiang and Wu (98)	China	cross-sectional	253	PSQI	>6	68.84 ± 11.11	4.75 ± 4.2	63.95 ± 11.18	UKPDS	20.77 ± 5.02	25.31 ± 4.10	27.88± 16.64	333.76 ± 327.31	NR	4
Zhang, Liu, Wang, Liu and Gu (99)	China	cross-sectional	127	PSQI	>5	66.38 ± 9.4	5.02 ± 3.6	NR	MDS-PD	NR	NR	NR	NR	2.44 ± 0.52	5
Zhang, Luo and Liao (100)	China	cross-sectional	95	PSQI	>6	NR	6.38 ± 4.38	61.51 ± 7.48	UKPDS	18.33 ± 3.40	20.75 ± 2.94	35.44 ± 17.05	NR	NR	4
Cui, Qing, Liu and Chen (101)	China	cross-sectional	138	PSQI	>4	NR	NR	NR	UKPDS	26.87 ± 1.83	NR	NR	NR	NR	5
song, Zhao, Li and Yin (102)	China	cross-sectional	40	PSQI	>7	63.85 ± 8.65	4.91 ± 3.51	NR	Diagnostic criteria for PD in China	NR	48.46 ± 23.25	NR	NR	2.84 ± 1.29	4
Song, Sun, Ma, Lu, Fan, Wang, Wang and Wang (103)	China	cross-sectional	80	PSQI	9 or above	66.82 ± 7.65	NR	NR	UKPDS	NR	NR	22.81 ± 4.19	NR	NR	5
Sun, Gao, Mo and Gong (104)	China	cross-sectional	56	PSQI	>6	55.77 ± 11.01	3.52 ± 2.86	51.86 ± 10.88	UKPDS	NR	NR	25.68 ± 13.96	NR	1.94	4
Lv, Wang, Zhu, Zhao and Guo (18)	China	cross-sectional	135	PSQI	>6	51.42 ± 14.63	2.35 ± 1.03	NR	Diagnostic criteria for PD in China	NR	NR	18.91 ± 5.21	NR	3.69 ± 0.53	5
Lu (105)	China	cross-sectional	70	PSQI	>7	64 ± 4	9 ± 4	NR	UKPDS	NR	NR	NR	NR	NR	4
Liu and Chen (106)	China	cross-sectional	82	PSQI	>5	64.21 ± 11.24	3.24 ± 1.45	NR	UKPDS	NR	NR	15.03 ± 8.61	NR	1.85 ± 0.97	4
Liu, Li, Fang, Qin and Wei (107)	China	cross-sectional	97	PSQI	>4	61.7 ± 9.2	2.1 ± 0.7	62.1 ± 9.7	PD criteria by NEDC	NR	NR	22.7 ± 8.9	432.3 ± 207.2	2.1 ± 0.6	4
Liu, Chou, Ma, Zhang, Wang and Gu (108)	China	cross-sectional	71	PSQI	>5	66.5 ± 8.66	6.11 ± 4.05	NR	UKPDS	22.03 ± 5.06	26.56 ± 3.10	NR	NR	2.4 ± 0.8	5
Liu, Wang, Wang, Mao and Wang (109)	China	cross-sectional	502	PSQI	>7	NR	NR	NR	UKPDS	NR	NR	NR	NR	NR	4
YU, PENG, LUO, HUANG and WANG (110)	China	cross-sectional	100	PSQI	>7	60.3 ± 8.26	4.24 ± 1.26	NR	Diagnostic criteria for PD in China	21.89 ± 4.70	NR	NR	NR	NR	4
Yi, Yu-Peng, Jiang-Ting, Jing-Yi, Qi-Xiong, Dan-Lei, Jing-Wei, Zhi-Juan, Yong-Jie, Zhe and Zheng (111)	China	cross-sectional	328	PSQI	9 or above	60.5 ± 10.1	4.78 ± 4.27	55.53 ± 10.34	MDS-PD	NR	25.19 ± 4.83	32.23 ± 17.30	575.78 ± 245.08	2.16 ± 0.99	5
Wang, Xiong, Chao, Zhuang, Li and Liu (112)	China	cross-sectional	938	PSQI	>5	NR	NR	NR	UKPDS	NR	NR	NR	NR	NR	5
Tang, Yang, Zhu, Gong, Sun, Chen, Guan, Yu, Wang, Zhang, Li, Ma and Wang (113)	China	cross-sectional	61	PSQI	>5	66.6 ± 6.10	4.5 ± 3.63	61.6 ± 7	UKPDS	26.38 ± 3.33	28.75 ± 2.22	22.63 ± 10	409.23 ± 249.56	2.45 ± 0.89	5
Song, Gu, An and Chan (114)	China	cohort	284	PSQI	>5	NR	NR	NR	UKPDS	NR	NR	NR	NR	NR	4
Shulman, Taback, Bean and Weiner (115)	United States	cross-sectional	99	PSQI	>5	67.4 ± 8	6.9 ± 5.7	NR	UKPDS	NR	NR	22 ± 9	NR	2.3 ± 0.8	6
Santos García, Cabo López, Labandeira Guerra, Yáñez Baña, Cimas Hernando, Paz González, Alonso Losada, Gonzalez Palmás, Cores Bartolomé and Martínez Miró (19)	Spain	cross-sectional	47	PSQI	>5	NR	NR	NR	UKPDS	NR	NR	NR	NR	NR	4
Sahebzadeh, Farsi Baf and Homam (116)	Iran	cross-sectional	120	PSQI	>5	65.9 ± 11.7	2.6 ± 2.7	NR	UKPDS	17.39 ± 5.20	NR	NR	NR	NR	6
Qiu, Gu, Liu and Li (117)	China	cross-sectional	101	PSQI	9 or above	61.25 ± 7.83	7.82 ± 1.34	NR	Diagnostic criteria for PD in China	NR	NR	NR	NR	NR	4
Qin, Li, Chen, Chen, Shi, Liu, Li, Xin and Gao (118)	China	cross-sectional	110	PSQI	>7	66.82 ± 8.23	3.67 ± 4.03	61.25 ± 10.38	MDS-PD	NR	NR	20.68 ± 18.02	447.32 ± 230.35	2.01 ± 0.87	4
Pandey, Bajaj, Wadhwa and Anand (17)	India	cross-sectional	100	PSQI	>5	59.2 ± 9.06	3.74 ± 3.67	55.5 ± 9.68	UKPDS	NR	NR	21.16 ± 6.98	367.53 ± 300.16	2.26 ± 0.64	4
Mahale, Yadav and Pal (119)	India	cross-sectional	156	PSQI	>5	52.47 ± 14.63	5.65 ± 4.64	48.49 ± 13.8	UKPDS	NR	27.73 ± 2.21	28.81 ± 10.77	602.73 ± 353.30	2.23 ± 0.51	4
Louter, van der Marck, Pevernagie, Munneke, Bloem and Overeem (120)	China	cross-sectional	153	PSQI	>5	65.9 ± 9.7	7.7 ± 5.7	NR	UKPDS	NR	NR	NR	468.4 ± 336.5	2.17	4
Lin, Chen, Lu, Huang, Weng, Yeh, Lin and Hung (121)	Taiwan	cross-sectional	225	PSQI	>5	65.7 ± 8.88	8.18 ± 5.2	57.5 ± 9.9	UKPDS	NR	NR	22.77 ± 10.8	425.84 ± 324.92	NR	5
Havlikova, van Dijk, Nagyova, Rosenberger, Middel, Dubayova, Gdovinova and Groothoff (122)	Slovak Republic	cross-sectional	93	PSQI	>4	68 ± 9.5	6.1 ± 5.9	NR	UKPDS	NR	NR	15.88 ± 10.43	NR	2 ± 1.2	5
Gao, Huang, Cai and Li (123)	China	cross-sectional	88	PSQI	>5	64.03 ± 10.5	5.41 ± 3.75	NR	MDS-PD	NR	25.59 ± 2.99	24.63 ± 7.58	510.85 ± 224.61	2.57 ± 1	4
Duncan, Khoo, Yarnall, O’Brien, Coleman, Brooks, Barker and Burn (124)	United Kingdom	cohort	158	PSQI	>5	66.5 ± 10.3	0.56 ± 0.49	NR	UKPDS	25.1 ± 3.6	28.6 ± 1.4	27.1 ± 12	NR	1.78	4
Ding, Zhu, Lu, Shen, Dai and Zhu (125)	China	cross-sectional	75	PSQI	9 or above	64.46 ± 6.46	NR	NR	PD criteria by NEDC	NR	NR	23.70 ± 3.81	NR	NR	4
Shafazand, Wallace, Arheart, Vargas, Luca, Moore, Katzen, Levin and Singer (126)	United States	cross-sectional	66	PSQI	>5	64.00 ± 10.00	NR	NR	UKPDS	NR	NR	NR	363.20 ± 255.30	NR	4
Skorvanek, Nagyova, Rosenberger, Krokavcova, Saeedian, Groothoff, Gdovinova and van Dijk (127)	Slovenská republika	cross-sectional	165	PSQI	>6	69.59 ± 8.56	6.93 ± 4.80	NR	UKPDS	NR	NR	30.11 ± 13.62	NR	2.41 ± 0.89	4
Shi, Guan, Gao, Huang and Wang (128)	China	cross-sectional	120	PSQI	9 or above	58.02 ± 8.56	5.48 ± 1.72	NR	Diagnostic criteria for PD in China	NR	NR	27.94 ± 8.98	NR	3.12 ± 1.22	4
Dong and Tan (129)	China	cross-sectional	92	PSQI	>7	69.77 ± 9.80	2.89 ± 4.00	NR	Diagnostic criteria for PD in China	NR	NR	24.84 ± 18.67	333.70 ± 134.80	2.27 ± 0.74	4
Chang, Fan, Chang and Wu (130)	Taiwan	cross-sectional	134	PDSS-2	>16	64.98 ± 9.19	7.86 ± 5.55	57.29 ± 10.66	UKPDS	NR	27.44 ± 1.88	23.91 ± 11.92	647.39 ± 491.49	1.43 ± 0.64	4
Shi (131)	China	cross-sectional	95	PDSS-2	>12	67.81 ± 5.93	4.1 ± 0.71	NR	Diagnostic criteria for PD in China	NR	NR	NR	NR	NR	4
Li, Yuan, Ye, Yuan, Gao and Hu (132)	China	cross-sectional	87	PDSS	<91	66.99 ± 7.18	5.75 ± 3.94	61.77 ± 7.84	Diagnostic criteria for PD in China	NR	27.58 ± 2.69	14.70 ± 5.25	490.78 ± 219.78	NR	4
Gui, Wang, Wu and Sun (133)	China	cross-sectional	178	PDSS	<91	61.81 ± 10.92	4.50 ± 3.48	56.53 ± 10.98	UKPDS	NR	26.61 ± 5.41	29.31 ± 14.66	441.26 ± 299.59	1.92 ± 0.75	4
Hu, Sun, Lan, Wang, Li and Zhong (134)	China	cross-sectional	207	PDSS-1	<6	61.29 ± 9.71	4.43 ± 4.74	NR	PD criteria by NEDC	16.97 ± 6.18	23.50 ± 5.45	NR	NR	2.34 ± 0.85	4
Niu and Gou (135)	China	cross-sectional	131	PDSS-1	<6	62.20 ± 8.00	2.27 ± 1.41	NR	Diagnostic criteria for PD in China	NR	NR	NR	NR	NR	4

NR, not reported; PSQI, Pittsburgh Sleep Quality Index; SD, standard deviation; H&Y stage, Hoehn and Yahr stage; UKPDS, UK Parkinson’s Disease Society Brain Bank Diagnostic Criteria; MDS-PD, Movement Disorder Society Clinical Diagnostic Criteria for Parkinson’s disease; PD criteria by NEDC, Parkinson’s diagnostic criteria established by Chinese Medical Association in the National Extrapyramidal Disorders Conference; MoCA, Montreal Cognitive Assessment; MMSE, Mini-Mental State Examination; UPDRS-3, The Unified Parkinson’s Disease Rating Scale part 3 (Motor Examination); LEDD, Levodopa equivalent daily dose; PSQI, Pittsburgh Sleep Quality Index; PDSS, Parkinson’s Disease Sleep Scale; PDSS-1, First item of Parkinson’s Disease Sleep Scale; PDSS-2, Parkinson’s disease sleep scale-2.

### 3.2 Pooled prevalence of poor sleep quality, PSQI global scores and PSQI component scores in Parkinson’s disease patients

**Figure 2:**
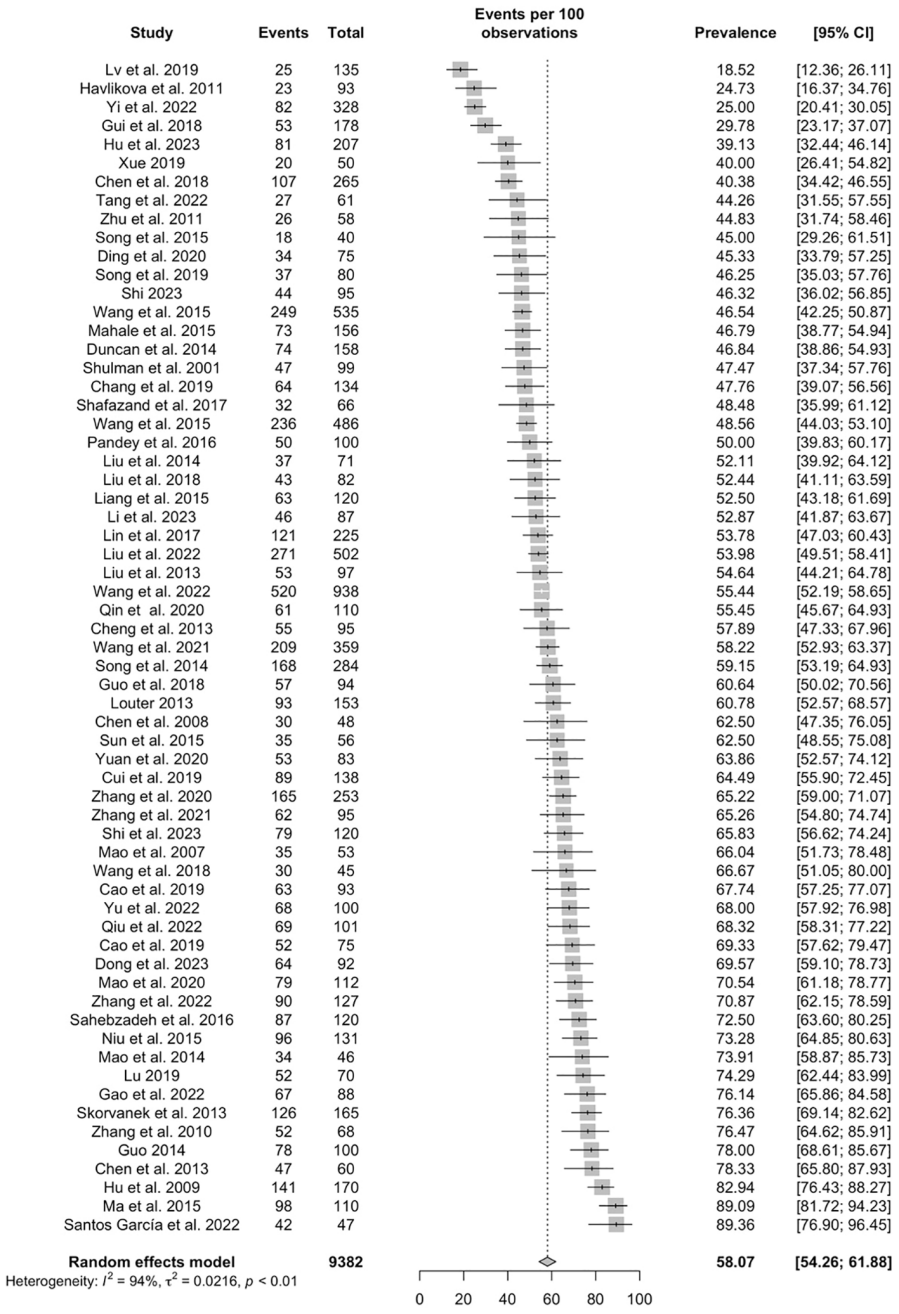
Forest plot of pooled prevalence of poor sleep quality in Parkinson’s disease patients.

Based on the 63 studies, the pooled prevalence of poor sleep quality in PD patients was 58.07% (95% CI: 54.26–61.88%) (**Figure 2**). As shown in **Table 2**, 28 studies with 3,369 participants reported PSQI global scores among PD patients, with a pooled PSQI total score of 8.6 (95% CI: 7.66-9.55). The seven PSQI sleep component scores were reported in 6 studies (N=540). The pooled PSQI component scores ranged from 0.73 (95% CI: 0.31-1.16) for “sleep medication use” to 1.76 (95% CI: 1.38-2.14) for “daytime dysfunction” (see **Table 2**).

**Table 2:** The pooled PSQI global and sleep component score in Parkinson’s disease patients.

Variables	Number of studies	Sample size	Pooled mean score (95% CI)	*I*2	*P* values
PSQI global score	28	3369	8.6101 (7.6664; 9.5538)	98.80%	< 0.0001
Subjective sleep quality	6	540	1.3010 (0.9834; 1.6187)	94.50%	< 0.0001
Sleep latency	6	540	1.3541 (0.9200; 1.7883)	97.40%	< 0.0001
Sleep duration	6	540	1.1770 (0.9375; 1.4164)	87.50%	< 0.0001
Habitual sleep efficiency	6	540	0.9736 (0.6394; 1.3078)	93.30%	< 0.0001
Sleep disturbance	6	540	1.4194 (1.2180; 1.6208)	91.80%	< 0.0001
Use of sleep medications	6	540	0.7347 (0.3085; 1.1609)	98.40%	< 0.0001
Daytime dysfunction	6	540	1.7561 (1.3769; 2.1354)	96.50%	< 0.0001

### 3.3 Subgroup and meta-regression analyses

Subgroup analysis by comparing means showed that study site was significantly associated with the prevalence of poor sleep quality (p < 0.01). Specifically, the highest prevalence of poor sleep quality in PD patients was reported in studies conducted in Europe & Central Asia (59.65% 95%CI: 37.63-81.68%), while the lowest figure was reported in North America (47.88%, 95%CI: 40.26–55.50%). There was also a significant subgroup difference in prevalence of poor sleep quality between countries by income (*P <*0.01): the highest prevalence was reported in upper-middle income countries (58.37%, 95%CI: 54.35–62.39%), while the lowest prevalence was reported in high income countries (56.40%, 95%CI: 40.51–72.28%). There were significant subgroup differences between PD diagnostic criteria (P < 0.01); the pooled prevalence of poor sleep quality was 59.04% (95% CI: 54.18-63.89%) in studies using the UK Parkinson’s Disease Society Brain Bank Diagnostic Criteria (UKPDS), 59.11% (95% CI: 40.65-77.57%) in studies using the Movement Disorder Society Clinical Diagnostic Criteria for Parkinson’s disease (MDS-PD), 57.20% (95% CI: 49.03-65.37%) in studies using the Diagnostic criteria of Chinese Parkinson’s Disease & Movement Disorders Society, Society for Neurology, Chinese Medical Association (Diagnostic criteria for PD in China) and 52.83% (95% CI: 42.44-63.22%) in studies using the diagnostic criteria for Parkinson’s disease established by the National Extrapyramidal Disorders Conference (PD criteria by NEDC). Furthermore, the pooled prevalence rates of poor sleep quality were 60.57% (95% CI: 50.23-70.91%) in inpatients, 53.66% (95% CI: 45.21-62.11%) in outpatients, and 62.86% (95% CI: 53.72-72.00%) in the mixed group. Moreover, there were significant differences (p < 0.01) between studies using different measures on sleep quality; the prevalence of poor sleep quality was 59.14% (95% CI: 55.19-63.09%) in studies using the PSQI, 40.96% (95% CI: 18.33-63.58%) in studies using the PDSS, 56.16% (95% CI: 22.69-89.62%) in studies using PDSS-1, and 47.16% (95% CI: 40.70-53.63%) in the studies using PDSS-2 (**Table 3**). In meta-regression analyses, onset age (β=0.0132, z=2.0704, p=0.0384) was positively associated with the prevalence of poor sleep quality. However, mean age, illness duration, levodopa equivalent daily dose, and The Unified Parkinson’s Disease Rating Scale part 3 (Motor Examination) score were not found to be statistically significant in the meta-regression (**Table 3**).

**Table 3:** Subgroup analyses and meta-regression analyses.

Subgroup analysis												
Subgroups		Categories		No. of studies		Prevalence (95%CI)		*I^2^*	*P* values within subgroups		*P* values across subgroups	
Geographic region		East Asia & Pacific		53		58.37% (54.35-62.39%)		94%	<0.01		<0.01	
		Europe & Central Asia		5		59.65% (37.61-81.68%)		97%	<0.01			
		South Asia		2		48.04% (41.93-54.16%)		0%	0.62			
		North America		2		47.88% (40.26-55.50%)		0%	0.9			
Countries by income		Upper middle income		53		58.37% (54.35-62.39%)		94%	<0.01		<0.01	
		High income		7		56.40% (40.51-72.28%)		96%	<0.01			
		Lower middle income		3		56.51% (40.51-72.51%)		91%	<0.01			
Study design		Cross-sectional		61		58.24% (54.32-62.16%)		94%	<0.01		<0.01	
		Cohort		2		53.29% (41.23-65.35%)		84%	0.01			
Diagnostic criteria		UKPDS		38		59.04% (54.18-63.89%)		94%	<0.01		<0.01	
		MDS-PD		5		59.11% (40.65-77.57%)		98%	<0.01			
		Diagnosticcriteria for PD in China		14		57.20% (49.03-65.37%)		93%	<0.01			
		PD criteria by NEDC		6		52.83% (42.44-63.22%)		83%	<0.01			
Cut-off value*		>4, >5, >6 and		41		59.61% (54.87-64.35%)		94%	<0.01		<0.01	
		>7 or above 7		16		57.93% (50.64-65.22%)		95%	<0.01			
Patient source		Mixed		15		62.86% (53.72-72.00%)		95%	<0.01		<0.01	
		Inpatients		3		60.57% (50.23-70.91%)		77%	0.01			
		Outpatients		12		53.66% (45.21-62.11%)		96%	<0.01			
Scale		PSQI		57		59.14% (55.19-63.09%)		94%	<0.01		<0.01	
		PDSS		2		40.96% (18.33-63.58%)		92%	<0.01			
		PDSS-1		2		56.16% (22.69-89.62%)		98%	<0.01			
		PDSS-2		2		47.16% (40.70-53.63%)		0%	0.83			

Meta-regression analyses							
Variables	Number of studies	Coefficient	Standard Error	95% Lower	95% Upper	z values	P values
Mean age	55	0.0076	0.0048	-0.0017	0.0169	1.5949	0.1107
Illness duration	51	0.0105	0.0124	-0.0139	0.0348	0.8415	0.4001
Male proportion	59	-0.1265	0.2418	-0.6005	0.3474	-0.5233	0.6008
Onset age	21	0.0132	0.0064	0.0007	0.0258	2.0704	**0.0384**
MoCA	16	-0.0047	0.0115	-0.0272	0.0178	-0.4075	0.6836
MMSE	19	-0.0044	0.0073	-0.0186	0.0099	-0.6044	0.5456
UPDRS-3	33	0.0068	0.0043	-0.0017	0.0153	1.5580	0.1192
LEDD mean	16	-0.0006	0.0004	-0.0014	0.0002	-1.5178	0.1291
H&YStage	31	-0.0126	0.0547	-0.1198	0.0946	-0.2304	0.8178
Study quality	63	-0.0682	0.0348	-0.1363	-0.0001	-1.9618	**0.0498**

*Only the study using the PSQI;H&Y stage: Hoehn and Yahr stage; UKPDS, UK Parkinson’s Disease Society Brain Bank Diagnostic Criteria; MDS-PD, Movement Disorder Society Clinical Diagnostic Criteria for Parkinson’s disease; PD criteria by NEDC, Parkinson’s diagnostic criteria established by the National Extrapyramidal Disorders Conference; MoCA, Montreal Cognitive Assessment; MMSE, Mini-Mental State Examination; UPDRS-3, The Unified Parkinson’s Disease Rating Scale part 3 (Motor Examination); H&Y stage, Hoehn and Yahr stage; LEDD, Levodopa equivalent daily dose; PSQI, Pittsburgh Sleep Quality Index; PDSS, Parkinson’s Disease Sleep Scale; PDSS-1, First item of Parkinson’s Disease Sleep Scale; PDSS-2, Parkinson’s disease sleep scale-2.

### 3.4 Publication bias and sensitivity analyses

The funnel plot assessment and Egger’s test value suggested no significant publication bias (Egger’s test t = 1.42, P= 0.1608) (See **Supplementary Figure S1**). Sensitivity analyses did not identify any outlying studies that could significantly change primary results (See **Supplementary Figure S2**). To avoid selection bias, no studies were excluded based solely on quality assessment. Sensitivity analysis confirmed that low-quality studies did not significantly alter the pooled prevalence estimates.

## 4 Discussion

To the best of our knowledge, this was the first systematic review and meta-analysis to examine the prevalence of poor sleep quality in patients with PD. Based on 63 studies, comprising 9,382 PD patients, the prevalence of poor sleep quality in patients with PD was 58.07% (95% CI: 54.26-61.88%). The factors associated with the prevalence of poor sleep quality included study region, income, diagnostic criteria, patient source and PD age of onset.

The pooled PSQI global score in this study was 8.61 (95% CI: 7.67-9.55), which was higher than the findings reported in most other population studies. For example, the global PSQI scores in a general population sample were 3.18 ± 2.28 in Japan (35), 5.00 ± 3.37 in Germany (36), 5.14 ± 3.90 in Spain (37), and 5.5 ± 2.8 in Singapore (38). The overall prevalence of poor sleep quality in PD patients was 58.07% (95% CI: 54.26-61.88%), which is substantially higher than most figures reported in other populations, including Singaporean working population (42.5%; 95% CI: 37.9-47.1%) (38), Chinese elderly population (35.9%; 95% CI: 30.6%-41.2%) (39), perinatal and postpartum women (54.2%; 95% CI: 47.9-60.5%) (24), medical students (55%; 95% CI 48.0%-62.0%) (40), patients with tinnitus (53.5%; 95% CI: 40.2-66.8%) (41), and patients with irritable bowel syndrome (37.6%; 95% CI: 31.4-44.3%) (42).

The high prevalence of poor sleep quality in PD patients may be attributed to the disease characteristics. Degenerative changes in the brain associated with the disease can directly impact sleep/wake mechanisms, leading to sleep disruptions (43). Additionally, movement difficulties, such as the inability to move around in bed, involuntary movements, dystonia, and pain caused by leg spasms, all of which can interfere with sleep maintenance (44). However, the overall prevalence of poor sleep quality in PD patients is lower than the rates reported in patients with chronic non-cancer pain (75.3%, 95% CI: 62.8-87.8%) (45), and hemodialysis patients (75.30%, 95% CI: 70.08-82.50%) (46). The prevalence is also lower than that in certain occupational groups with high work demands, such as nurses (61.00%, 95% CI: 55.8-66.1%) (47) and military personnel (69.00%, 95% CI: 62.33-75.30%) (25), who often experience high stress, shift work, long work hours, high burnout, and exposure to trauma (48).

In subgroup analyses, we found that the overall prevalence of poor sleep quality in PD patients was significantly lower in North America (47.88%, 95% CI: 40.26-55.50%) and high-income countries (56.40%, 95% CI: 40.51-72.28%) compared to other regions and countries. The burden of PD appears to be greater in low-income areas, which may contribute to poorer sleep quality (49, 50). On the other hand, higher-income countries may have access to better healthcare facilities, resources, and support, which can indirectly lower the risk of poor sleep quality PD patients (51). Greater healthcare resources in high-income countries may lead to more effective management of symptoms in PD patients in these countries, and thereby improving their sleep quality.

The overall prevalence of poor sleep quality was significantly higher among inpatients (60.57%, 95% CI: 50.23-70.91%) compared to outpatients (53.66%, 95% CI: 45.21-62.11%), which may be attributed to the severity of PD. Hospitalized patients usually suffer from a more advanced stage of the disease, necessitating closer medical monitoring and treatment (52). Certain symptoms, such as tremors, muscle stiffness, and difficulties with motor control, can all contribute to disrupted sleep (53, 54). Furthermore, changes in the environment such as being hospitalized or separated from their families and regular living environment can also contribute to a decline in sleep quality (55, 56). Consequently, hospitalized PD patients are more likely to encounter sleep problems.

In the subgroup analyses, the pooled prevalence of poor sleep quality in studies using the MDS-PD (59.11%, 95% CI: 40.65-77.57%) was significantly higher than those using the diagnostic PD criteria by NEDC (52.83%, 95% CI: 42.44-63.22%). Notably, certain studies had applied the UK Parkinson’s Disease Society Brain Bank (UKPDS) criteria and the Parkinson’s diagnostic criteria established by Chinese Medical Association (CMA) in the National Extrapyramidal Disorders Conference (NEDC), both of which differ from the Movement Disorder Society Clinical Diagnostic Criteria for Parkinson’s disease(MDS-PD) in terms of diagnostic strictness and clinical application. Such diagnostic variations represent a key source of heterogeneity: the MDS-PD incorporates advanced biomarkers and updated clinical features to maximize specificity (23), whereas the UKPDS relies primarily on classic motor symptoms with established high sensitivity but lower specificity for early PD. The CMA guidelines adapt international standards to regional practices, potentially introducing operational variability, and NEDC (1984) lacks contemporary validation with possible under-identification of non-motor phenotypes (57, 58). Such methodological heterogeneity can directly influence prevalence estimates, as stricter criteria (e.g., MDS-PD) may capture patients with more advanced pathology and higher comorbidity burden, while broader criteria (e.g., NEDC/UKPDS) can include borderline cases with milder sleep disturbances. Consequently, the observed prevalence differences likely reflect both true biological variability and diagnostic threshold effects, necessitating cautious interpretation of cross-criteria study comparisons.

Subgroup analyses also revealed significantly higher pooled prevalence rates in cross-sectional studies (58.24%, 95% CI: 54.32-62.16%) compared to cohort studies (53.29%, 95% CI: 41.23-65.35%). It should be noted that only a small number of cohort studies (n = 2) were included, which might have resulted in less reliable estimates. Similarly, the higher prevalence observed in studies using the PSQI might partially reflect selection bias, due to the small number of studies using the PDSS (n = 2), PDSS-1 (n = 2), and PDSS-2 (n=2). Therefore, the findings of these subgroup analysis are tentative.

In meta-regression analyses, the age of onset of PD was positively associated with prevalence of poor sleep quality. Previous studies found that gender and age of onset are significant factors influencing the clinical phenotype of PD, affecting both motor symptoms and various non-motor symptoms (59). Patients with late-onset PD tend to experience more frequent non-motor symptoms, including cognitive dysfunction, autonomic dysfunction, and sleep disturbances (60, 61). A study conducted in China on non-motor symptoms in PD found positive correlations between age, daily levodopa dose, and Non-Motor Symptoms Scale (NMSS) scores in patients with late-onset PD. However, these correlations were not observed in those with early-onset PD, suggesting that age and daily levodopa dose may play a more significant role in the severity of non-motor symptoms in patients with late-onset PD compared to those with early-onset PD (62). Several brainstem nuclei are potentially involved in the mechanisms underlying non-motor symptoms such as gastrointestinal regulation, pain perception, emotion control, and sleep-wake cycles (63, 64). The presence of Lewy bodies, which are composed of abnormal α-synuclein and are found outside the dopaminergic neurons of the midbrain, probably contribute to the pathology of non-motor manifestations in PD (63, 65, 66). Different distribution and severity of central nervous system lesions and pathophysiological course of PD between patients with early-onset and late-onset PD may be crucial factors influencing the non-motor features (e.g., sleep quality) associated with the disease (62).

Overall, our meta-analysis indicates that patients with PD have a high prevalence of poor sleep quality. Sleep problems are closely associated with mental health (67), and patients with PD often experience psychological stress (68), anxiety and depression (69, 70). Therefore, maintaining good sleep quality plays a crucial role in sustaining emotional and mental stability and enhancing the mental well-being and quality of life for patients (71). Cognitive decline and difficulties with concentration are common challenges faced by PD patients (72, 73). Prioritizing sleep quality in patients with PD is essential for managing symptoms, enhancing mental health, and preserving cognitive functioning. By optimizing the sleep environment, establishing healthy sleep habits, and utilizing medications or other treatments, when necessary, the sleep quality of patients with PD can be improved. Moreover, meta-regression showed that study quality was negatively associated with sleep quality in PD patients. Thus, lower study quality may lead to underestimation of sleep problems in PD patients. Improving the methodological rigor of studies may help to more accurately assess the severity of sleep quality problems (74).

Strengths of this meta-analysis include high number of included studies and large sample size, and the utilization of comprehensive analytical methods, such as subgroup and meta-regression analyses, to identify factors associated with poor sleep quality. Nonetheless, several limitations should be noted. First, despite use of sophisticated statistical methods, heterogeneity still remained in the subgroup analyses, as is expected in meta-analyses of epidemiological surveys, as pointed out in previous studies (25, 47). Second, certain factors related to poor sleep quality, such as living environment, psychiatric comorbidities, and educational background, were not included in most studies. Third, wide confidence intervals for subgroups might be related to small sample sizes or the use of different PD diagnostic criteria in some studies. Fourth, the overrepresentation of Chinese studies might introduce bias that could affect the generalizability of the findings to other populations (75). Fifth, while our analysis identified key correlates of poor sleep quality, the preponderance of cross-sectional data precluded any causal conclusions. Longitudinal studies are warranted to explore the temporal relationships, particularly between sleep deterioration and PD progression trajectories. Furthermore, significant heterogeneity in PD diagnostic criteria (e.g., MDS-PD vs. UKPDS vs. CMA vs. NEDC) could result in methodological variability. Although this reflects real-world clinical practice, differences in diagnostic sensitivity/specificity might influence pooled prevalence estimates and subgroup comparability. Future meta-analyses would benefit from standardized application of contemporary diagnostic frameworks of PD.

In conclusion, poor sleep quality is common among patients with PD, particularly in lower middle-income countries and Europe & Central Asia regions. To mitigate the adverse effects of poor sleep quality in PD patients, regular monitoring of sleep quality and sleep hygiene is crucial, particularly in high-risk subgroups. Additionally, further studies of sleep quality and PD patients in low- and middle-income countries are warranted, as well as prospective cohort studies to clarify the causality between poor sleep quality and other factors.

## Data Availability

The original contributions presented in the study are included in the article/**Supplementary Material**. Further inquiries can be directed to the corresponding authors.

## Supplementary material

Supplementary Table 1 Quality assessment of included studies.

Supplementary Figure 1 Funnel plot of pooled prevalence of poor sleep quality in Parkinson’s disease patients.

Supplementary Figure 2 Sensitivity analysis of pooled prevalence of poor sleep quality in Parkinson’s disease patients.
