# Supplementary material for: Poor sleep quality among patients with Parkinson’s disease: a meta-analysis and systematic review
Source: Front Psychiatry. 2025 Jul 16;16:1606743. doi: 10.3389/fpsyt.2025.1606743 (PMC12307349; doi:10.3389/fpsyt.2025.1606743)
Supplement: Supplementary Table 1 — Quality assessment of included studies. [file SupplementaryFile1.docx]

**Supplementary materials**

Table S1. Quality assessment of included studies

Figure S1. Funnel plot of pooled prevalence of poor sleep quality in Parkinson’s disease patients

Figure S2. Sensitivity analysis of pooled prevalence of poor sleep quality in Parkinson’s disease patients

**Table S1.** Quality assessment of included studies

| Authors and citation No. | Target population is clearly defined? | probability sampling OR entire population surveyed | Is the response rate ≥80% | Are non-responders clearly described? | Do the characteristics of respondents match the target population? | Were data collection methods standardized? | Were validated criteria used to diagnose poor sleep quality？ | Are the prevalence estimates given with confidence intervals and detailed by subgroups (if applicable)? | Total scores |
| --- | --- | --- | --- | --- | --- | --- | --- | --- | --- |
| Ma and Ren ^76^ | 1 | 0 | 0 | 0 | 1 | 1 | 1 | 1 | 5 |
| Chen, Zhang, Zhou, Yang and Feng ^77^ | 1 | 0 | 0 | 0 | 1 | 1 | 1 | 0 | 4 |
| Chen, Hu, Han, Zhang and Zhang ^78^ | 1 | 0 | 0 | 0 | 1 | 1 | 1 | 0 | 4 |
| Chen, Hong and Chen ^79^ | 1 | 0 | 0 | 0 | 1 | 1 | 1 | 0 | 4 |
| Guo, Yu, Zuo, Liu, Li, Pu, Hu, Lian, Wang, Yu, Jin, Zhu and Zhang ^80^ | 1 | 0 | 0 | 0 | 1 | 1 | 1 | 0 | 4 |
| Guo ^81^ | 1 | 0 | 0 | 0 | 1 | 1 | 1 | 0 | 4 |
| Yuan, Hu, Zhao, Wang, Zhang and Li ^82^ | 1 | 0 | 0 | 0 | 1 | 1 | 1 | 0 | 4 |
| Xue ^83^ | 1 | 0 | 0 | 0 | 1 | 1 | 1 | 0 | 4 |
| Hu and Zhang ^84^ | 1 | 0 | 0 | 0 | 1 | 1 | 1 | 0 | 4 |
| Cheng, Yu and Wu ^85^ | 1 | 0 | 0 | 0 | 1 | 1 | 1 | 0 | 4 |
| Wang, Feng, Gu, Liu and Chen ^86^ | 1 | 0 | 0 | 0 | 1 | 1 | 1 | 1 | 5 |
| Wang, Feng, Gu, Liu, Zhang and Chen ^87^ | 1 | 0 | 0 | 0 | 1 | 1 | 1 | 1 | 5 |
| Wang and Wang ^88^ | 1 | 0 | 0 | 0 | 1 | 1 | 1 | 1 | 5 |
| Wang, Sun, Ma, Shi, Li, Huang, Hu and Zheng ^89^ | 1 | 0 | 0 | 0 | 1 | 1 | 1 | 0 | 4 |
| Mao and Zhang ^90^ | 1 | 0 | 0 | 0 | 1 | 1 | 1 | 0 | 4 |
| Mao, Dai and Liu ^91^ | 1 | 0 | 0 | 0 | 1 | 1 | 1 | 0 | 4 |
| Mao and Saimaiti ^92^ | 1 | 0 | 0 | 0 | 1 | 0 | 1 | 0 | 3 |
| Liang, Cui, Wu, Yu and Chen ^93^ | 1 | 0 | 0 | 0 | 1 | 1 | 1 | 0 | 4 |
| Zhu, Zheng and Yang ^94^ | 1 | 0 | 0 | 0 | 1 | 1 | 1 | 1 | 5 |
| Cao, Liu, Ma, Chen, Ren, Zhang and Xu ^95^ | 1 | 0 | 0 | 0 | 1 | 1 | 1 | 0 | 4 |
| Cao, Yu, Su and Guo ^96^ | 1 | 0 | 0 | 0 | 1 | 1 | 1 | 0 | 4 |
| Zhang, Gao and Wei ^97^ | 1 | 0 | 0 | 0 | 1 | 1 | 1 | 0 | 4 |
| Zhang, Zhang, Zhu, Jiang and Wu ^98^ | 1 | 0 | 0 | 0 | 1 | 1 | 1 | 0 | 4 |
| Zhang, Liu, Wang, Liu and Gu ^99^ | 1 | 0 | 0 | 0 | 1 | 1 | 1 | 1 | 5 |

**Table S1.** Quality assessment of included studies. (continued)

| Authors and publication year | Target population is clearly defined? | probability sampling OR entire population surveyed | Is the response rate ≥80% | Are non-responders clearly described? | Do the characteristics of respondents match the target population? | Were data collection methods standardized? | Were validated criteria used to diagnose poor sleep quality？ | Are the prevalence estimates given with confidence intervals and detailed by subgroups (if applicable)? | Total scores |
| --- | --- | --- | --- | --- | --- | --- | --- | --- | --- |
| Zhang, Luo and Liao ^100^ | 1 | 0 | 0 | 0 | 1 | 1 | 1 | 0 | 4 |
| Cui, Qing, Liu and Chen ^101^ | 1 | 0 | 0 | 0 | 1 | 1 | 1 | 1 | 5 |
| song, Zhao, Li and Yin ^102^ | 1 | 0 | 0 | 0 | 1 | 1 | 1 | 0 | 4 |
| Song, Sun, Ma, Lu, Fan, Wang, Wang and Wang ^103^ | 1 | 0 | 0 | 0 | 1 | 1 | 1 | 1 | 5 |
| Sun, Gao, Mo and Gong ^104^ | 1 | 0 | 0 | 0 | 1 | 1 | 1 | 0 | 4 |
| Lv, Wang, Zhu, Zhao and Guo ^18^ | 1 | 0 | 0 | 0 | 1 | 1 | 1 | 1 | 5 |
| Lu ^105^ | 1 | 0 | 0 | 0 | 1 | 1 | 1 | 0 | 4 |
| Liu and Chen ^106^ | 1 | 0 | 0 | 0 | 1 | 1 | 1 | 0 | 4 |
| Liu, Li, Fang, Qin and Wei ^107^ | 1 | 0 | 0 | 0 | 1 | 1 | 1 | 0 | 4 |
| Liu, Chou, Ma, Zhang, Wang and Gu ^108^ | 1 | 0 | 0 | 0 | 1 | 1 | 1 | 1 | 5 |
| Liu, Wang, Wang, Mao and Wang ^109^ | 1 | 0 | 0 | 0 | 1 | 1 | 1 | 0 | 4 |
| YU, PENG, LUO, HUANG and WANG ^110^ | 1 | 0 | 0 | 0 | 1 | 1 | 1 | 0 | 4 |
| Yi, Yu-Peng, Jiang-Ting, Jing-Yi, Qi-Xiong, Dan-Lei, Jing-Wei, Zhi-Juan, Yong-Jie, Zhe and Zheng ^111^ | 1 | 0 | 0 | 1 | 1 | 1 | 1 | 0 | 5 |
| Wang, Xiong, Chao, Zhuang, Li and Liu ^112^ | 1 | 0 | 0 | 0 | 1 | 1 | 1 | 1 | 5 |
| Tang, Yang, Zhu, Gong, Sun, Chen, Guan, Yu, Wang, Zhang, Li, Ma and Wang ^113^ | 1 | 0 | 0 | 0 | 1 | 1 | 1 | 1 | 5 |
| Song, Gu, An and Chan ^114^ | 1 | 0 | 0 | 0 | 1 | 1 | 1 | 0 | 4 |
| Shulman, Taback, Bean and Weiner ^115^ | 1 | 0 | 1 | 1 | 1 | 1 | 1 | 0 | 6 |

| Authors and publication year | Target population is clearly defined? | probability sampling OR entire population surveyed | Is the response rate ≥80% | Are non-responders clearly described? | Do the characteristics of respondents match the target population? | Were data collection methods standardized? | Were validated criteria used to diagnose poor sleep quality？ | Are the prevalence estimates given with confidence intervals and detailed by subgroups (if applicable)? | Total scores |
| --- | --- | --- | --- | --- | --- | --- | --- | --- | --- |
| Santos García, Cabo López, Labandeira Guerra, Yáñez Baña, Cimas Hernando, Paz González, Alonso Losada, Gonzalez Palmás, Cores Bartolomé and Martínez Miró ^19^ | 1 | 0 | 0 | 0 | 1 | 1 | 1 | 0 | 4 |
| Sahebzadeh, Farsi Baf and Homam ^116^ | 1 | 1 | 0 | 0 | 1 | 1 | 1 | 1 | 6 |
| Qiu, Gu, Liu and Li ^117^ | 1 | 0 | 0 | 0 | 1 | 1 | 1 | 0 | 4 |
| Qin, Li, Chen, Chen, Shi, Liu, Li, Xin and Gao ^118^ | 1 | 0 | 0 | 0 | 1 | 1 | 1 | 0 | 4 |
| Pandey, Bajaj, Wadhwa and Anand ^17^ | 1 | 0 | 0 | 0 | 1 | 1 | 1 | 0 | 4 |
| Mahale, Yadav and Pal ^119^ | 1 | 0 | 0 | 0 | 1 | 1 | 1 | 0 | 4 |
| Louter, van der Marck, Pevernagie, Munneke, Bloem and Overeem ^120^ | 1 | 0 | 0 | 0 | 1 | 1 | 1 | 0 | 4 |
| Lin, Chen, Lu, Huang, Weng, Yeh, Lin and Hung ^121^ | 1 | 0 | 0 | 0 | 1 | 1 | 1 | 1 | 5 |
| Havlikova, van Dijk, Nagyova, Rosenberger, Middel, Dubayova, Gdovinova and Groothoff ^122^ | 1 | 0 | 1 | 0 | 1 | 1 | 1 | 0 | 5 |
| Gao, Huang, Cai and Li ^123^ | 1 | 0 | 0 | 0 | 1 | 1 | 1 | 0 | 4 |
| Duncan, Khoo, Yarnall, O'Brien, Coleman, Brooks, Barker and Burn ^124^ | 1 | 0 | 0 | 0 | 1 | 1 | 1 | 0 | 4 |
| Ding, Zhu, Lu, Shen, Dai and Zhu ^125^ | 1 | 0 | 0 | 0 | 1 | 1 | 1 | 0 | 4 |
| Shafazand, Wallace, Arheart, Vargas, Luca, Moore, Katzen, Levin and Singer ^126^ | 1 | 0 | 0 | 0 | 1 | 1 | 1 | 0 | 4 |

**Table S1.** Quality assessment of included studies. (continued)

**Table S1.** Quality assessment of included studies. (continued)

| Authors and publication year | Target population is clearly defined? | probability sampling OR entire population surveyed | Is the response rate ≥80% | Are non-responders clearly described? | Do the characteristics of respondents match the target population? | Were data collection methods standardized? | Were validated criteria used to diagnose poor sleep quality？ | Are the prevalence estimates given with confidence intervals and detailed by subgroups (if applicable)? | Total scores |
| --- | --- | --- | --- | --- | --- | --- | --- | --- | --- |
| Skorvanek, Nagyova, Rosenberger, Krokavcova, Saeedian, Groothoff, Gdovinova and van Dijk ^127^ | 1 | 0 | 0 | 0 | 1 | 1 | 1 | 0 | 4 |
| Shi, Guan, Gao, Huang and Wang ^128^ | 1 | 0 | 0 | 0 | 1 | 1 | 1 | 0 | 4 |
| Dong and Tan ^129^ | 1 | 0 | 0 | 0 | 1 | 1 | 1 | 0 | 4 |
| Chang, Fan, Chang and Wu ^130^ | 1 | 0 | 0 | 0 | 1 | 1 | 1 | 0 | 4 |
| Shi ^131^ | 1 | 0 | 0 | 0 | 1 | 1 | 1 | 0 | 4 |
| Li, Yuan, Ye, Yuan, Gao and Hu ^132^ | 1 | 0 | 0 | 0 | 1 | 1 | 1 | 0 | 4 |
| Gui, Wang, Wu and Sun ^133^ | 1 | 0 | 0 | 0 | 1 | 1 | 1 | 0 | 4 |
| Hu, Sun, Lan, Wang, Li and Zhong ^134^ | 1 | 0 | 0 | 0 | 1 | 1 | 1 | 0 | 4 |
| Niu and Gou ^135^ | 1 | 0 | 0 | 0 | 1 | 1 | 1 | 0 | 4 |

**Figure S1.** Funnel plot of pooled prevalence of poor sleep quality in Parkinson’s disease patients


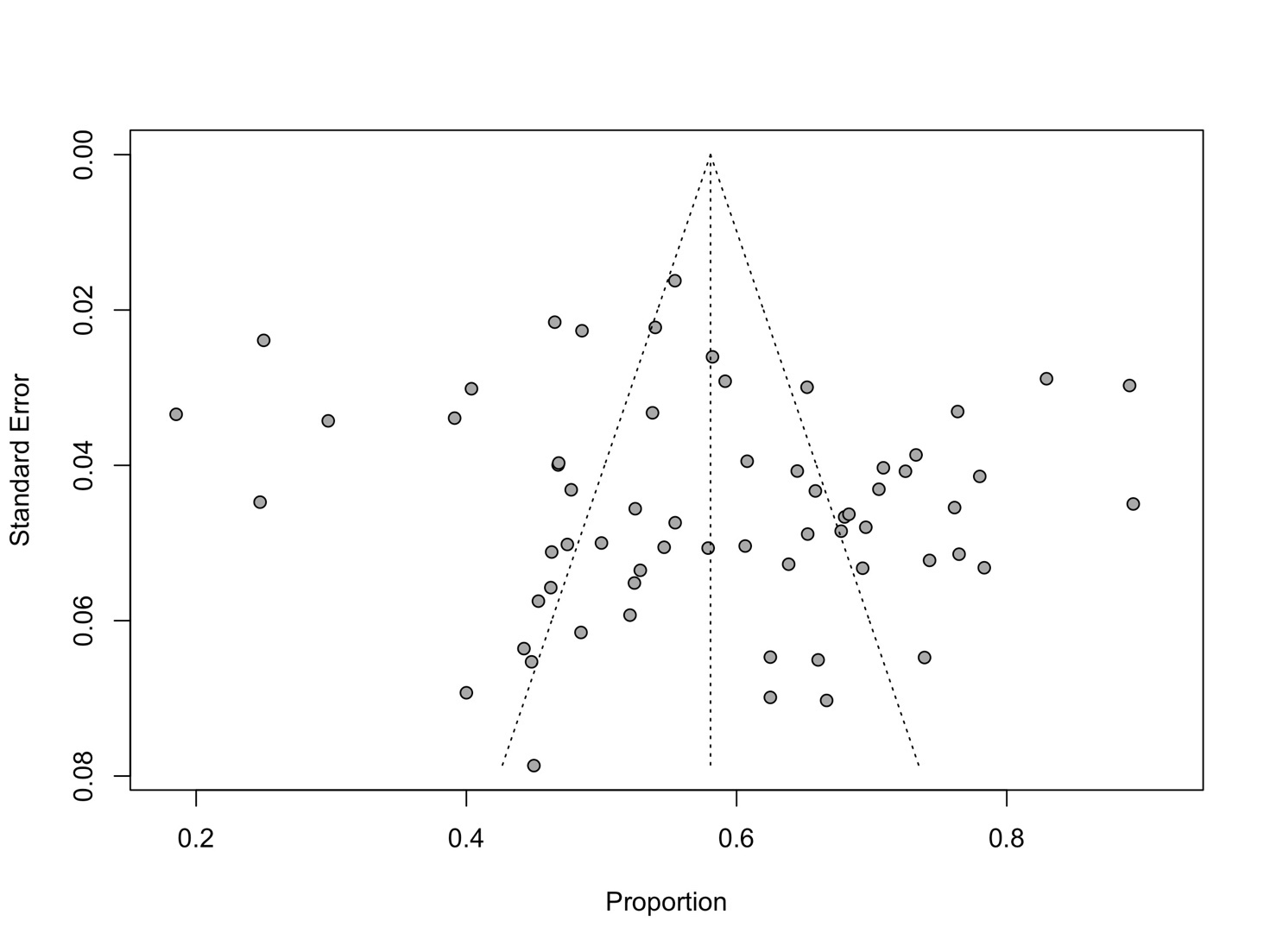


Funnel plot of pooled prevalence of poor sleep quality from 63 included studies (Egger’s test *t* = 1.42, *P*= 0.1608)

**Figure S2.** Sensitivity analysis of pooled prevalence of poor sleep quality in Parkinson’s disease patients

**
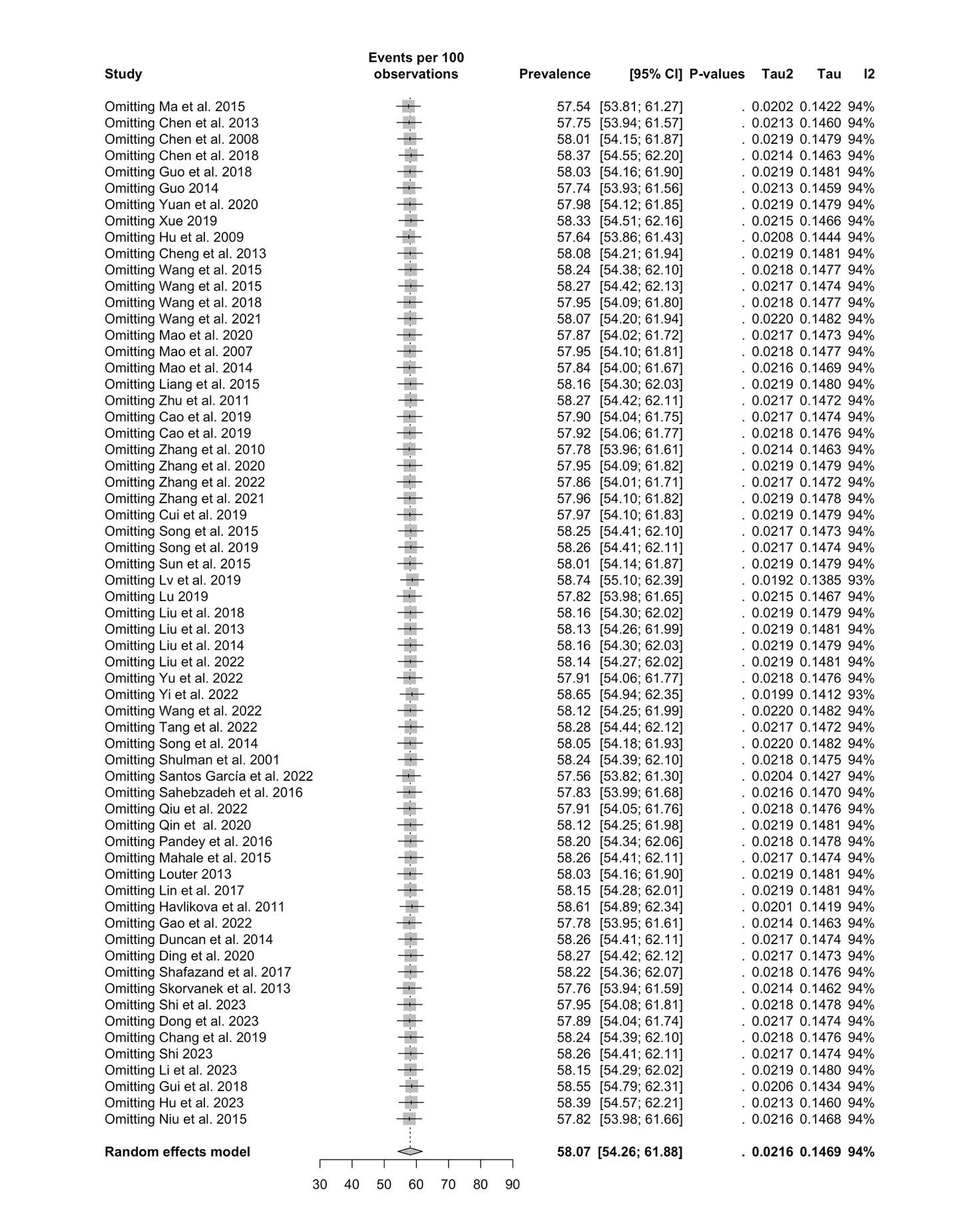
**
